# StructScan3D v1: A First RGB-D Dataset for Indoor Building Elements Segmentation and BIM Modeling

**DOI:** 10.3390/s25113461

**Published:** 2025-05-30

**Authors:** Ishraq Rached, Rafika Hajji, Tania Landes, Rashid Haffadi

**Affiliations:** 1 College of Geomatic Sciences and Surveying Engineering, Institute of Agronomy and Veterinary Medicine, Rabat 6202, Morocco; r.hajji@iav.ac.ma; 2 ICube Laboratory UMR 7357, Photogrammetry and Geomatics Group, National Institute of Applied Sciences (INSA Strasbourg), 24, Boulevard de la Victoire, 67084 Strasbourg, France; tania.landes@insa-strasbourg.fr; 3 GEOPTIMA, B4, Med El Amraoui Street, Corner of Sebou Street, Office 4, Kenitra, Morocco; rashidhaffadi@gmail.com

**Keywords:** semantic segmentation, RGB-D, Kinect Azure, BIM, structural elements, dataset

## Abstract

The integration of computer vision and deep learning into Building Information Modeling (BIM) workflows has created a growing need for structured datasets that enable the semantic segmentation of indoor building elements. This paper presents StructScan3D v1, the first version of an RGB-D dataset specifically designed to facilitate the automated segmentation and modeling of architectural and structural components. Captured using the Kinect Azure sensor, StructScan3D v1 comprises 2594 annotated frames from diverse indoor environments, including residential and office spaces. The dataset focuses on six key building elements: walls, floors, ceilings, windows, doors, and miscellaneous objects. To establish a benchmark for indoor RGB-D semantic segmentation, we evaluate D-Former, a transformer-based model that leverages self-attention mechanisms for enhanced spatial understanding. Additionally, we compare its performance against state-of-the-art models such as Gemini and TokenFusion, providing a comprehensive analysis of segmentation accuracy. Experimental results show that D-Former achieves a mean Intersection over Union (mIoU) of 67.5%, demonstrating strong segmentation capabilities despite challenges like occlusions and depth variations. As an evolving dataset, StructScan3D v1 lays the foundation for future expansions, including increased scene diversity and refined annotations. By bridging the gap between deep learning-driven segmentation and real-world BIM applications, this dataset provides researchers and practitioners with a valuable resource for advancing indoor scene reconstruction, robotics, and augmented reality.

## 1. Introduction

The progress of computer vision and machine learning technologies has increased the demand for high-quality datasets, which are characterized by good accuracy, completeness, correctly labeled data, and density, particularly in the realm of RGB-D (Red, Green, Blue, and Depth) imagery. In recent years, RGB-D imagery has proven a good level of relevance and efficiency in a wide range of applications, including robotics, autonomous navigation [1,2,3], augmented reality, and indoor scene reconstruction [4,5,6]. Among these, one of the most impactful applications lies in Building Information Modeling (BIM). By leveraging RGB-D data, BIM enables the representation of structured elements of building environments, supporting tasks such as design, analysis, construction monitoring, and maintenance [7,8].

BIM is increasingly recognized as a transformative approach for reducing costs and improving efficiency in the construction industry. By integrating spatial, temporal, and semantic information within a unified digital environment, BIM enables key applications such as automated design validation, space optimization, energy performance analysis, and predictive maintenance. Recent research [9] has demonstrated that semantic segmentation plays a central role in automating the generation of BIM models from 3D scene data. In this context, semantic segmentation of indoor environments serves as a crucial bridge between raw sensor data and structured BIM representations, allowing for automated processes that replace traditional, labor-intensive workflows. Adopting RGB-D sensors in this context holds immense potential thanks to portability and cost-effectiveness. These sensors capture both visual and depth data, enhancing the ability to identify and classify structural elements under varying lighting conditions and complex indoor geometries. However, despite their capabilities, there is a lack of datasets tailored to address the specific challenges of BIM creation, such as comprehensive structural annotations, scene diversity, and temporal continuity, as provided with RGB-D video sequences.

Existing RGB-D datasets, such as NYU Depth v2 [10] and Matterport3D [11], have laid the foundation for advancements in semantic segmentation. However, they are often limited in addressing the specific needs of BIM creation. Most of these datasets focus on general object detection, such as everyday items like furniture (chairs and tables) or appliances (microwaves and televisions), or sparse single-frame annotations, overlooking critical structural elements such as walls, floors, ceilings, doors, and windows, which are essential for accurate BIM modeling and architectural analysis. Inconsistent variety in scenes, lack of annotations that account for changes over time, and biases caused by the specific features of sensors further limit their use in practical architectural applications.

To address these challenges, we introduce a new RGB-D dataset captured using the Kinect Azure sensor, specifically designed to support automated BIM creation. This dataset offers high-resolution RGB images and accurate depth information, enabling the precise analysis of structural elements across diverse indoor environments. It includes a combination of annotated video sequences and individual frames, providing both spatial and temporal data representations, with a focus on key structural categories such as walls, windows, floors, ceilings, doors, and other elements.

The dataset was acquired using the Kinect Azure sensor, which recorded high-resolution MKV video files containing synchronized RGB images and corresponding depth information. The Kinect Azure’s advanced sensing capabilities, including improved depth accuracy and a wide field of view, were leveraged to capture detailed architectural features crucial for BIM applications. This has enabled the inclusion of intricate structural details in the dataset, which are essential for accurate indoor modeling and BIM creation.

Although the specifications of the Kinect Azure suggest that it is well suited for this task, further experiments and analyses are needed to fully demonstrate its effectiveness in improving the accuracy of automatic BIM generation. These future studies will solidify the dataset’s role in supporting the development of robust computer vision models tailored for architectural applications.

In summary, this paper presents StructScan3D v1, a novel RGB-D dataset specifically developed to support semantic segmentation tasks in the context of BIM and indoor scene understanding, such as automated as-built modeling, spatial reasoning for robotics, and AR/VR applications. This first version focuses on essential architectural elements (walls, floors, ceilings, windows, doors, and others).

The main contributions of this work are as follows:

1. The creation of the first annotated RGB-D dataset dedicated to the segmentation of BIM-relevant structural elements in indoor environments.
2. The implementation of a real-world acquisition protocol using a low-cost RGB-D sensor, under realistic conditions including ambient lighting variability, structural occlusions, and diverse indoor layouts.
3. The inclusion of temporally coherent RGB-D image sequences, enabling research on spatio-temporal semantic segmentation and supporting model development for sequence-based indoor understanding.

This paper is organized as follows: Section 2 reviews related work, focusing on existing RGB-D datasets, sensors, and RGB-D semantic segmentation, and identifies the gaps within them. Section 3 describes the materials and methods used in creating the StructScan3D v1 dataset, including data acquisition, annotation processes and preprocessing techniques. Section 4 presents the experimental results and baseline evaluation setup, highlighting both quantitative metrics, as well as qualitative evaluations through visual segmentation comparison. Finally, Section 5 concludes the paper by summarizing the contributions of the proposed dataset and outlines potential directions for future research in the field.

## 2. Related Work

The use of RGB-D data has significantly transformed numerous computer vision applications, particularly in the domains of semantic segmentation, 3D reconstruction, and scene understanding. This section provides an overview of the key components relevant to the development of RGB-D datasets, including sensor technologies, existing datasets, annotation methodologies, and recent advancements in RGB-D semantic segmentation.

### 2.1. RGB-D Sensors

RGB-D sensors have significantly contributed to progress in scene understanding, enabling applications in 3D reconstruction and scene categorization. Notable RGB-D sensors, such as the Microsoft Kinect and Intel RealSense, differ in their technical specifications, including depth resolution, color quality, and power requirements, which influence their applicability across various settings [12].

Kinect v1 and v2, for example, are among the earliest widely used RGB-D sensors. Kinect v1 utilizes structured light, projecting an infrared pattern to generate depth maps, but exhibits depth quantization artifacts. Kinect v2, which employs a time-of-flight mechanism, provides more accurate depth measurements and better captures fine-grained details, although it has limitations with dark or reflective surfaces. Kinect v2 also requires significant power and is bulkier compared to more compact options like Intel RealSense [12].

Intel RealSense has been designed for portable devices, offering the advantages of lower power consumption and reasonably accurate depth maps within shorter ranges (up to 3.5 m). However, it lacks the depth precision offered by the Kinect v2 sensor at larger distances. This trade-off makes RealSense a suitable choice for mobile applications, but it may be less suitable for tasks that require high-precision depth information. Moreover, RGB-D datasets, such as NYU Depth v2 [10] and SUN RGB-D [13], are based on multiple sensors to enhance generalizability across different environments, thus supporting broader scene understanding applications.

RGB-D sensors often face limitations when used in environments with insufficient lighting, highly reflective surfaces (e.g., mirrors and glass), or complex geometries, leading to depth inaccuracies or missing data [14]. To mitigate these issues during the acquisition of the StructScan3D dataset, we carefully selected acquisition conditions: we ensured sufficient ambient lighting, avoided scanning directly in front of mirrors or glass surfaces, and controlled the sensor trajectory to maintain optimal range and angle. While this does not eliminate sensor limitations, these precautions reduced depth noise and missing regions, ensuring better data quality for semantic segmentation and indoor modeling.

### 2.2. RGB-D Datasets

RGB-D datasets are essential for various computer vision applications, providing both color (RGB) and depth (D) information, which can be used to understand the 3D structure of objects and environments. These datasets have been widely used for tasks such as monocular depth estimation, object segmentation, and human activity recognition [15]. In the domain of RGB-D semantic segmentation, the extensive literature covers the use of RGB-D datasets, deep learning methodologies, and their applications in tasks such as indoor BIM [8]. Semantic segmentation in RGB-D data combines RGB imagery with depth information to classify pixels, leading to enhanced scene understanding and object recognition, especially in complex indoor environments [8].

RGB-D datasets typically include RGB images which are the standard images captured by optical cameras for providing color information. They also produce depth maps which provide information about the range distance between camera and object. Depth can be captured using various sensors like LiDAR (Light Detection And Ranging), structured light, or stereo cameras. The combination of these two types of data allows for more accurate and detailed analysis. For example, in robotics, RGB-D data can help a robot navigate and interact with its environment more effectively by understanding the 3D layout and discover objects [16].

In augmented reality, this data can be used to overlay digital content onto the physical world in a way that accurately aligns with the real-world objects [17]. Table 1 reports the main used RGB-D datasets in the literature with regards to their limitations. While datasets like NYU Depth v2 [10] and SUN RGB-D [13] offer comprehensive class annotations, they lack sequential data for spatio-temporal modeling. On the other hand, large-scale datasets such as ScanNet [18] and Matterport3D [11] provide extensive scene coverage but suffer from sparse labeling or a focus on panoramic images, limiting their applicability for pixel-wise segmentation tasks. Synthetic datasets like SceneNet RGB-D [19] and InteriorNet [20], despite their size and variety, face challenges in generalization due to domain gaps with real-world data. These observations underscore the need for a robust real-world RGB-D dataset that offers dense annotations and temporal dynamics, which are often lacking in existing datasets. Indicators such as the number of annotated frames, the inclusion of sequential data for spatio-temporal modeling, and the diversity of structural elements can serve as benchmarks to highlight these gaps. For instance, as shown in the comparative table, existing datasets like NYU Depth v2 and SUN RGB-D provide limited temporal annotations or focus on single-frame segmentation, leaving a need for datasets capable of supporting sequence-based segmentation tasks.

### 2.3. RGB-D Annotations

The annotation process for RGB-D datasets, particularly within indoor environments, has evolved significantly, beginning with foundational datasets like NYU Depth v2. This dataset includes over 400,000 pairs of RGB and depth frames, with 1449 frames manually labeled across 40 semantic classes. The annotations cover indoor scenes, capturing diverse real-world conditions [10,22]. The annotation methodology involves several key aspects:

**Object Labels and Categories:** NYU Depth v2 provides detailed labels for a broad set of object categories, from larger structures like walls and floors to smaller, specific items such as books and TVs, aiming to represent a realistic distribution of objects in indoor spaces. This comprehensive labeling requires considerable manual input, particularly to capture diverse object classes in typical home and office settings [10].

**Depth Utilization:** The dataset leverages depth information to enhance annotation accuracy, applying techniques like Conditional Random Fields (CRFs) [23] and Multi-scale Convolutional Networks to refine object boundaries in complex scenes. Objects with consistent depth features, such as ceilings and floors, are particularly suited to depth-based differentiation, illustrating the utility of depth data in disambiguating objects based on their spatial position and relative distance [23].

**Temporal Smoothing in Video Sequences**: NYU Depth v2 also includes sequences without extensive frame-by-frame ground truth labels. However, techniques such as temporal super-pixel smoothing are employed in a post-processing phase to reduce flickering effects, and helping therefore to enhance temporal consistency in object labels across video frames [22].

Further extending these annotation approaches, the Matterport3D dataset incorporates panoramic RGB-D images with detailed 3D surface reconstructions and instance-level semantic segmentations. This dataset captures entire buildings, including examples such as office complexes and residential spaces, and provides globally aligned views for comprehensive scene analysis, supporting tasks such as 3D object detection, room layout estimation, and indoor navigation. Matterport3D’s annotation process combines crowdsourcing and machine learning techniques to generate high-quality, instance-level labels across varied viewpoints, ensuring its applicability to diverse computer vision challenges [11].

Despite these advances, a major limitation remains in the lack of fully annotated sequential RGB-D datasets suitable for spatio-temporal model training. For example, tasks like modeling scene dynamics, temporal consistency in segmentation across frames, and action recognition require datasets with dense labeling over time-series sequences. Existing datasets, such as NYU Depth v2 and Matterport3D, are largely single-frame focused or lack dense annotations across time, which restricts their application to tasks that demand an integrated spatial and temporal understanding of scenes.

### 2.4. RGB-D Semantic Segmentation

Research in RGB-D semantic segmentation has progressed from earlier, feature-based methods to sophisticated, deep learning architectures that leverage the combined strengths of RGB and depth data [24]. Initially, feature-based approaches relied on handcrafted features, such as texture descriptors and geometric cues derived from depth data, which were combined with RGB color information to classify pixels and understand scene structure. While effective to an extent, these methods were limited by the scope of predefined features and struggled with complex indoor scenes that contain significant variation in lighting, occlusion, and object positioning [10]. With the emerging of deep learning, however, convolutional neural networks (CNNs) and their variants transformed the field by automatically learning high-level features directly from data. These advanced architectures can capture intricate patterns in both RGB and depth modalities, allowing for more accurate and context-aware segmentation.

Recently, research has expanded to include multi-modal fusion, multi-scale processing, and even spatio-temporal models that utilize sequential RGB-D data, representing a substantial leap in the capability to segment and understand indoor environments comprehensively.

### 2.5. Synthesis

The current gap lies in the lack of RGB-D datasets annotated for time-series data, which is essential for training spatio-temporal models. Most existing RGB-D datasets lack sequential annotations that capture temporal dependencies across frames. This gap limits the ability of segmentation models to leverage temporal coherence, which is beneficial in real-world applications like robot navigation, dynamic scene understanding, and autonomous driving. By introducing an RGB-D dataset with annotated sequences, research can move toward creating models that understand and predict changes over time, a critical advancement for understanding in real-world settings. The literature on RGB-D semantic segmentation in indoor environments reflects the field’s progression from early, feature-based methods to advanced deep learning approaches that capitalize on the synergy of RGB and depth data. While recent methods have yielded significant improvements in segmentation accuracy, challenges remain in areas such as temporal modeling and cross-sensor generalization. The emerging research on spatio-temporal models and multimodal fusion techniques holds promise for addressing these challenges, advancing RGB-D segmentation for practical applications in robotics, augmented reality, and BIM.

## 3. Materials and Methods

This section details the process and tools used to create, organize, annotate, and preprocess the dataset. It also describes the evaluation methodology employed to establish a baseline and validate the quality of the dataset for semantic segmentation tasks. The workflow consists of five main steps: data collection, data storage, data annotation, data processing, and dataset splitting.

### 3.1. Data Collection

The dataset was collected using the Kinect Azure sensor (Microsoft, https://azure.microsoft.com/fr-fr/products/kinect-dk, accessed on 1 January 2021). Its time-of-flight mechanism ensured the detailed capture of architectural features. The sensor record synchronized RGB and depth streams, which facilitates the precise alignment of the two modalities.

#### 3.1.1. Scene Selection

**Figure 1:**
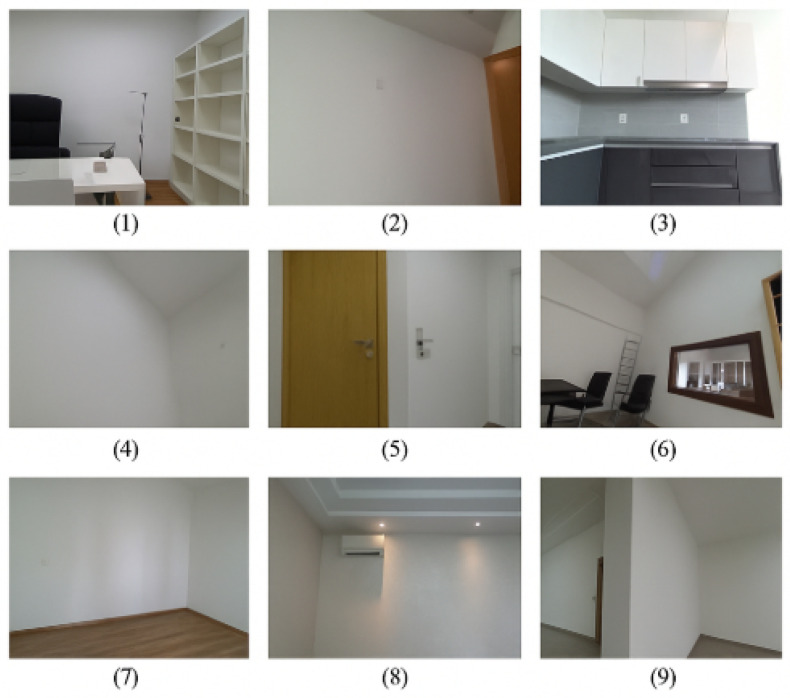
Examples of RGB frames from different indoor scenes captured in StructScan3D v1: (1) an office scene with desk, chair, and shelving units; (2) a room with large planar wall surface and indirect lighting; (3) a kitchen area with reflective cabinet surfaces; (4) a corner wall zone under low-light conditions; (5) a door transition area with complex geometry and occlusion; (6) a meeting room with chairs, windows, and mixed lighting; (7) a nearly empty bedroom with wooden flooring; (8) a living space with an air conditioning unit and ceiling lights; and (9) a corridor scene with occlusion and partial visibility.

To ensure diversity and representativeness, nine distinct indoor scenes were selected, which encompass a wide range of architectural environments with different complexities. Representative examples of these indoor scenes are illustrated in Figure 1, showcasing the diversity of spatial layouts and structural elements captured in the StructScan3D v1 dataset.

**Residential apartments** shown in Figure 1: Featuring a mix of open and confined spaces, with varied wall textures, furniture arrangements, and natural lighting.

**Office spaces:** Including modular layouts, glass partitions, and dynamic lighting conditions from natural and artificial sources.

**Figure 2:**
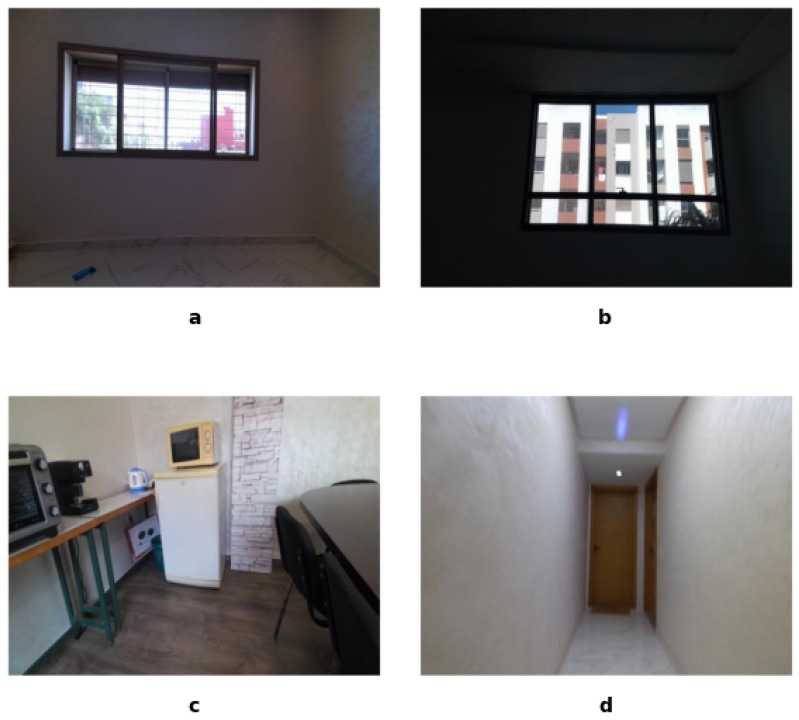
Examples of scene diversity in the dataset: (**a**) material properties, (**b**) lighting conditions, (**c**) occlusions, and (**d**) spatial layout.

The scenes in the StructScan3D v1 dataset were carefully selected to highlight variations that enhance its applicability for robust model training and testing. Examples of variations in material properties are shown in Figure 2a, while acquisitions under a variety of lighting conditions are represented in Figure 2b. Occlusions, caused by furniture and objects, are shown in Figure 2c, and the diversity of the spatial layout, such as narrow hallways, is illustrated in Figure 2d. These examples emphasize the real-world complexities captured in the dataset, ensuring that it is well suited for training and evaluating semantic segmentation models in diverse indoor environments.

#### 3.1.2. Data Acquisition Protocol

**Figure 3:**
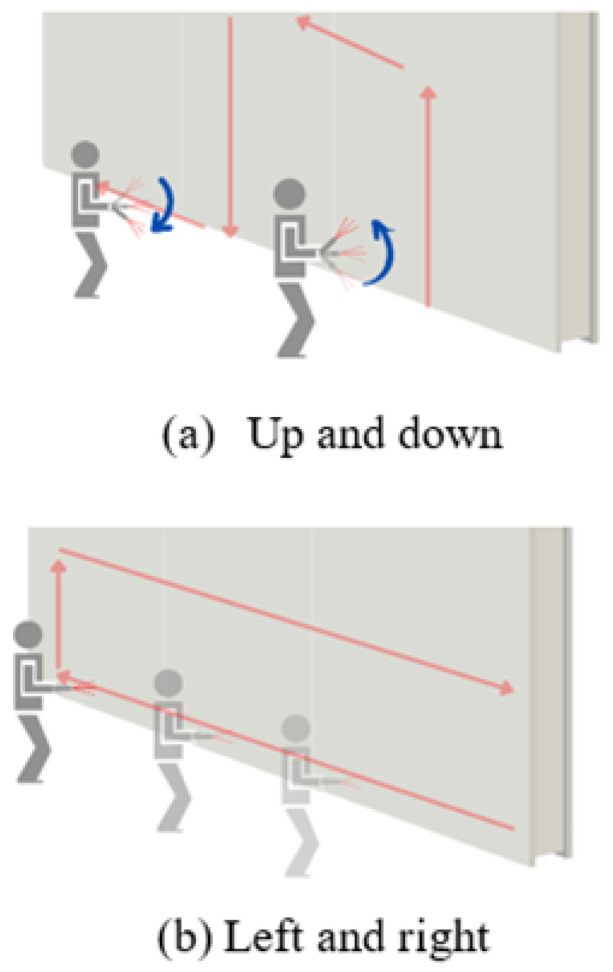
Illustration of the RGB-D data acquisition protocol used in StructScan3D v1. (**a**) Vertical (up and down) scanning motion to capture wall height and ceiling detail. (**b**) Horizontal (left to right) sweep to ensure full coverage along walls and furniture lines.

Data were captured in Matroska (MKV) video format, leveraging the advanced capabilities of the Kinect Azure sensor. The acquisition protocol was meticulously designed to ensure comprehensive coverage of each scene. For clarity, each large scene was subdivided into smaller, manageable sub-scenes to facilitate systematic data collection. The protocol adhered to the following guidelines proposed in [25]:

**Unidirectional Movement Protocol:** To ensure consistent coverage and alignment, the operator followed a structured acquisition protocol based on vertical and horizontal camera sweeps as illustrated in Figure 3. The up-and-down motion (Figure 3a) captured ceiling and floor boundaries, while the left-to-right motion (Figure 3b) ensured wall continuity and object alignment. The protocol was applied across all recorded scenes to support stable and complete RGB-D data collection under realistic indoor conditions. [8,25]

**Minimized Occlusion:** By maintaining a steady sensor orientation relative to the scene, the protocol minimized occlusions and maximized the visibility of structural features.

**Multiple Passes in Complex Areas:** For complex scenes with intricate details or heavy occlusion, the operator performed multiple passes to ensure all elements were adequately recorded.

This structured acquisition process enhanced the dataset’s quality, making it well-suited for applications requiring precise architectural modeling and spatio-temporal analysis.

### 3.2. Data Storage

To enable effective analysis and modeling, the recorded video data was systematically converted and organized for seamless navigation and scalability. The raw MKV videos were converted into individual image frames using *Open3D*, a library designed for 3D data processing that provides tools for handling point clouds, RGB-D images, and geometric operations [26] and custom *Python* scripts. The process involves two key steps:

**MKV Reader Script:** Extract synchronized RGB and depth frames from the MKV files, ensuring temporal alignment between the two modalities.

**Frame Extraction Script:** Parse the video streams into sequential images, generating a dataset suitable for both spatial and temporal analyses.

This conversion process ensured the dataset maintained the integrity of the original recordings while offering flexibility for analytical workflows such as semantic segmentation, object detection, and time-series modeling. The extracted images were systematically organized to facilitate easy navigation and management. Each image was assigned a unique filename using the format “scene_subscene_image_number”. For example, the filename scene1_2_15 indicates that the image is the 15th frame from the second sub-scene of scene 1.

This convention ensures the following:

**Traceability:** Users can easily identify the scene and sub-scene associated with each image.

**Scalability:** The organization system can accommodate additional scenes or sub-scenes without requiring significant adjustments.

This approach provides a robust foundation for dataset management and exploration, supporting diverse research and application needs. To provide an overview of the dataset’s structure, metadata were compiled summarizing the number of sub-scenes and total images for each scene. The metadata table facilitates efficient exploration and analysis by offering a high-level understanding of the dataset’s composition. Table 2 below summarizes the dataset’s structure:

The data annotation process was meticulously designed to ensure accurate and comprehensive semantic segmentation labels for each frame, focusing on both geometric precision and level of detail. To achieve high-quality annotations, the process combined automated methods for initial labeling with manual refinement to correct errors and capture finer structural features. Geometric quality was ensured by leveraging precise depth data from the Kinect Azure sensor, allowing accurate alignment between RGB and depth information. The level of detail was addressed by annotating each frame across six structural classes—walls, floors, ceilings, doors, windows, and others—ensuring that even smaller and intricate elements were consistently labeled. This dual focus on geometric accuracy and detailed labeling makes the dataset robust for advanced semantic segmentation and scene reconstruction tasks.

The annotation process consisted of two primary steps:

**Semi-Automatic Annotation:** Initial segmentation masks were generated using Roboflow, a platform powered by pretrained models. This automated step streamlined the annotation process by handling straightforward elements, such as walls and floors.

**Manual Refinement:** To ensure accuracy and consistency, annotations were manually reviewed and adjusted, particularly for complex scenarios like occluded elements (e.g., windows partially hidden by furniture) and overlapping objects (e.g., doorframes intersecting with walls). This step addresses edge cases where automatic tools were less reliable, ensuring precise boundary definitions for intricate details.

This two-step approach ensures high-quality annotations that capture both geometric accuracy and detailed labeling, essential for robust semantic segmentation.

### 3.3. Data Preprocessing

The preprocessing of RGB and depth data is crucial to ensure consistency and compatibility across the dataset. The objective is to standardize the input formats, enhance data quality, and prepare the dataset for training machine learning models.

**Depth Processing:** Depth maps underwent several preprocessing steps to improve their usability and ensure compatibility with machine learning frameworks:

**Resize:** All depth maps were resized to a resolution of 480 × 640 pixels, maintaining uniformity across the dataset.

**Auto-Orientation:** Depth images were rotated automatically to correct any misalignment during acquisition, ensuring consistent orientation.

**Normalization:** Depth values were normalized to reduce sensor-specific noise, standardize the depth range, and enhance model performance.

These steps ensured that the depth data retained its structural details while adhering to a consistent format for seamless integration into segmentation workflows. Common challenges during this process, such as depth inconsistencies caused by sensor noise, misalignment between RGB and depth frames, and artifacts in depth maps (e.g., missing or distorted values near reflective or transparent surfaces), were carefully mitigated. By addressing these typical pitfalls, the preprocessing pipeline was optimized to provide clean, reliable depth data suitable for accurate segmentation and robust model performance.

**RGB Processing:** RGB images were processed to standardize their format and improve compatibility with machine learning models:

**Resize:** All RGB images were resized to a resolution of 480 × 640 pixels to match the depth data and meet model input requirements.

**Normalization:** Pixel intensity values were normalized to align with the input specifications of deep learning frameworks.

The preprocessing of both RGB and depth data ensures that the dataset adhered to consistent standards, enabling efficient and accurate training of semantic segmentation models.

### 3.4. Dataset Splitting

To prepare the dataset for training and evaluation, it was divided into two subsets: a training set comprising approximately 70% of the dataset, with 1900 images from six scenes (two residential and four office), and a testing set representing the remaining 30%, with 600 images from three scenes (one residential and two office). This division ensures a balanced distribution of scene types for robust model training and evaluation. Given the sequential nature of the data, random splitting was avoided to preserve the time-series structure. Instead, entire sub-scenes were allocated to either the training or testing set, maintaining logical scene progression to enable the learning and evaluation of spatio-temporal dependencies. This structured splitting strategy ensures that models trained on the dataset can generalize effectively while preserving the integrity of temporal relationships.

For the test set, a deliberate choice was made to include one residential scene to capture the challenges and complexity typically found in home environments. Residential scenes, which often involve varied furniture arrangements, architectural features, and intricate spatial relationships, are more complex compared to office environments. Including this type of scene in the test set ensures that the model is evaluated under more challenging conditions, helping to assess its ability to generalize across different real-world indoor settings. These scenes, along with the office environments, were annotated and segmented using the semi-automatic process followed by manual refinement, ensuring high-quality annotations that represent typical indoor structures and variability.

**Figure 4:**
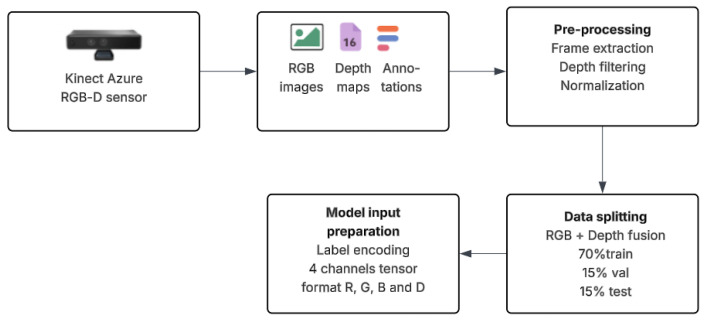
StructScan3D v1 dataset-processing pipeline.

To summarize, Figure 4 illustrates the StructScan3D v1 dataset pipeline, from initial acquisition to model-ready input preparation. RGB-D data was collected using a handheld Azure Kinect sensor in real indoor environments.

### 3.5. Dataset Characteristics

The dataset’s composition and features are summarized in the table below, highlighting its diversity, structure, and applicability to indoor scene understanding tasks.

To ensure that the training dataset adequately represents the objects of interest, careful consideration was given to selecting scenes that encompass a diverse range of structural elements and environmental conditions. As summarized in Table 3, the annotated classes include walls, floors, ceilings, doors, windows, and other structural components, which are critical for achieving accurate semantic segmentation in indoor environments.

The training dataset was designed to balance class distribution while maintaining diversity in scene contexts. It includes a mix of residential and office spaces, chosen to capture a wide variety of textures, materials, lighting conditions, and spatial layouts. This diversity ensures that the trained models can generalize effectively to new environments while addressing the specific requirements of applications in BIM generation and indoor scene reconstruction.

Additionally, the dataset addresses the challenge of underrepresented classes, such as windows and doors, by ensuring their adequate inclusion in the training set. This approach mitigates potential class imbalances and enhances the model’s ability to accurately segment these elements. Overall, this strategic design of the training dataset supports robust model training and improves segmentation performance across diverse and complex scenarios.

The following section presents the experimental setup, implementation details, and evaluation results, demonstrating the effectiveness of the dataset and the D-Former model in supporting robust semantic segmentation tasks.

## 4. Experiments and Results

This section presents the implementation details and evaluation results of a baseline model trained on the proposed RGB-D dataset. The experiments specifically focus on validating the dataset’s utility for semantic segmentation of indoor structural elements, such as walls, floors, and ceilings. The goal is to assess the dataset’s effectiveness in improving segmentation performance and its suitability for targeted applications, including BIM creation and indoor scene reconstruction.

### 4.1. Baseline Model

To establish a baseline for evaluating the dataset, we utilized the D-Former model [27], a state-of-the-art transformer-based architecture specifically designed for semantic segmentation of RGB-D data. The primary objective was to assess the dataset’s suitability for accurately capturing indoor building elements, such as walls, floors, and ceilings, while addressing the inherent complexities of indoor environments.

Transformer-based architectures, such as D-Former, have gained prominence in computer vision for their ability to model long-range dependencies through self-attention mechanisms [28,29]. This capability makes them particularly effective in tasks requiring a global understanding of spatial relationships, such as semantic segmentation. Unlike traditional convolutional networks, D-Former combines a lightweight robust architecture with multi-scale feature extraction, enabling efficient handling of diverse spatial features and complex layouts typical of indoor scenes [8].

These characteristics make D-Former an ideal choice for leveraging the depth and RGB data in our dataset, enabling precise semantic segmentation of structural elements such as walls, floors, and ceilings. This decision aligns with recent advancements in segmentation tasks, where transformer-based models have demonstrated superior performance over convolutional networks in both accuracy and generalization [27].

### 4.2. Implementation

The implementation of the D-Former model followed a structured and systematic approach, where the key steps of the implementation process are as follows:

**Input data:** The dataset consisted of RGB-D inputs (four channels: three for RGB and one for depth) and their corresponding ground truth segmentation masks. Preprocessing steps included resizing the RGB and depth images to a resolution of 480 × 640 pixels for consistency across the dataset.

**Model architecture:** D-Former-base, a smaller and more efficient variant of the D-Former family, is particularly well-suited for applications requiring a balance between computational efficiency and segmentation accuracy. The encoder leverages a SegFormer-base transformer backbone to extract rich, multi-scale features from RGB-D inputs, effectively capturing both global context and fine details. A lightweight decoder then aggregates these multi-scale features to produce high-resolution segmentation maps, ensuring fine-grained pixel-wise classification. This choice of D-Former-S allows for the efficient processing of the StructScan3D dataset while maintaining robust performance, particularly in segmenting.

**Environment setup:** The environment setup for implementing the D-Former model involved creating a *Python* virtual environment using *Conda* to ensure compatibility and isolation. *Python 3.10* was used, and the latest *PyTorch version (2.1.2)* with *CUDA 11.8* support was installed for accelerated computations on *NVIDIA GPUs*. The required dependencies, including *torchvision*, *torchaudio*, and *mmcv*, were installed to support the transformer-based architecture and its associated functionalities. Additional libraries such as *tqdm*, *opencv-python*, *scipy*, *tensorboardX*, *tabulate*, *easydict*, *ftfy*, and *regex* were also installed to facilitate data handling, training visualization, and efficient computation. This streamlined setup ensured a robust environment for training and evaluating the D-Former model on the StructScan3D dataset.

**Training configuration:** The model was trained using the proposed StructScan3D dataset, with a batch size and learning rate optimized for the lightweight D-Former Small architecture. Key hyperparameters, including the optimizer, loss function, and learning rate scheduler, were selected based on prior experiments [8] to ensure efficient convergence.

**Figure 5:**
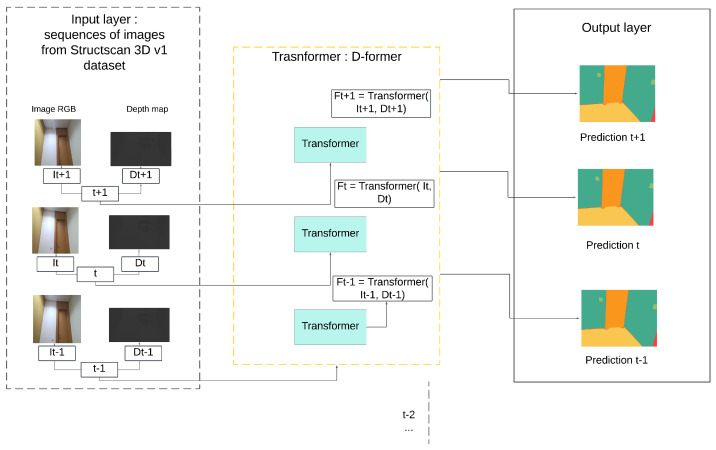
Overview of the D-Former processing pipeline applied to StructScan3D v1.

The processing flow is illustrated in Figure 5, showing how RGB-D input from StructScan3D v1 is encoded and segmented frame by frame using the D-Former architecture. Each frame is independently encoded, and the extracted features are used to generate semantic segmentation maps.

These implementation details reflect the practical considerations and optimization strategies undertaken to adapt D-Former to the StructScan3D dataset. They highlight its suitability for indoor semantic segmentation tasks by ensuring robust learning from RGB-D data and maintaining computational efficiency, key for real-world applications such as BIM and robotics.

### 4.3. Results of Semantic Segmentation Evaluation

The evaluation of the proposed StructScan3D dataset and the performance of the D-Former model is based on two key analyses: quantitative analysis and qualitative analysis. These complementary approaches provide a comprehensive understanding of the model’s effectiveness and the dataset’s utility for semantic segmentation tasks in complex indoor environments.

#### Quantitative Analysis

To assess the performance of the D-Former model trained on the StructScan3D dataset, we employed four primary metrics: Mean Intersection over Union (mIoU), F1 Score, Pixel Accuracy, and Training and Validation Loss. These metrics, widely recognized in semantic segmentation tasks, offer complementary perspectives to evaluate model performance comprehensively.

### 4.4. Evaluation Metrics

To evaluate model performance on semantic segmentation tasks, we adopt standard metrics including Mean Intersection over Union (mIoU), F1 Score, and Pixel Accuracy.

**Mean Intersection over Union (mIoU)** provides a comprehensive evaluation by considering true positives, false positives, and false negatives across all classes. It is particularly suitable for complex indoor scenes, as it balances over-segmentation and under-segmentation. The metric is defined as

(1) $$\mathrm{mIoU}=\frac{1}{N}\sum_{i=1}^{N}\frac{TP_{i}}{TP_{i}+FP_{i}+FN_{i}}$$

where

- $TP_{i}$: True positives for class *i*;
- $FP_{i}$: False positives for class *i*;
- $FN_{i}$: False negatives for class *i*;
- *N*: Total number of classes.

**F1 Score** reflects the harmonic mean of precision and recall. It provides insights into the model’s ability to balance false positives and false negatives, especially for small or occluded structures. The F1 Score for a specific class is given by

(2) $$F1=2\times \frac{\mathrm{Precision}\times \mathrm{Recall}}{\mathrm{Precision}+\mathrm{Recall}}$$

where precision and recall are defined as

(3) $$\mathrm{Precision}=\frac{TP_{i}}{TP_{i}+FP_{i}}$$

(4) $$\mathrm{Recall}=\frac{TP_{i}}{TP_{i}+FN_{i}}$$

**Pixel Accuracy** measures the ratio of correctly classified pixels to the total number of pixels:

(5) $$\mathrm{Pixel}\;\mathrm{Accuracy}=\frac{\mathrm{Total}\;\mathrm{Correctly}\;\mathrm{Predicted}\;\mathrm{Pixels}}{\mathrm{Total}\;\mathrm{Pixels}}$$

All values are computed from pixel-level comparisons between the predicted segmentation masks and ground truth annotations on a per-class basis. These counts are aggregated across the validation or test set, and the metrics (mIoU, F1 Score, and Pixel Accuracy) are then calculated accordingly.

**Figure 6:**
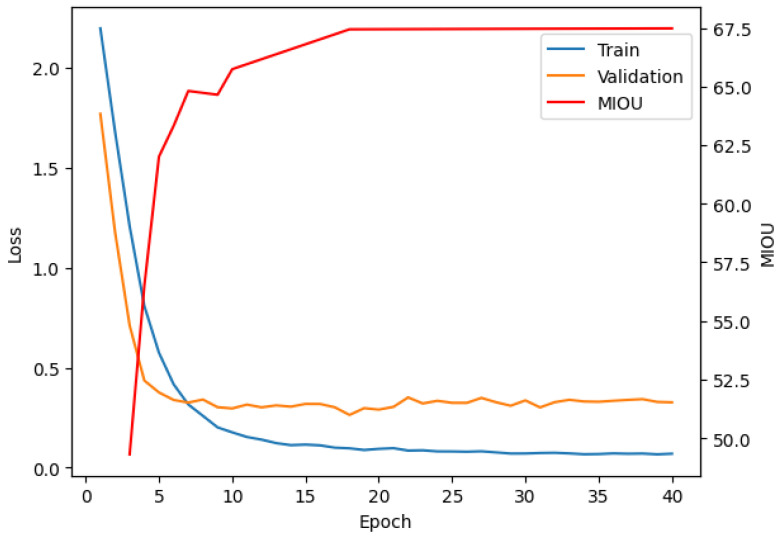
Training progression of the D-Former model on StructScan3D v1. The plot shows training loss (blue), validation loss (orange), and mean IoU (red).

The results, illustrated in Figure 6, highlight the progression of training loss, validation loss, and mIoU during the training of the D-Former model on the StructScan3D dataset. The training and validation losses (blue and orange lines) are computed using the cross-entropy loss function, while the mIoU (red line) is calculated using standard per-class IoU across the validation set. The training loss (blue line) demonstrates a consistent decline, showcasing the model’s iterative refinement in minimizing discrepancies between predicted outputs and ground truth labels. The validation loss (orange line) stabilizes after approximately 15 epochs, suggesting strong generalization to unseen data and minimal overfitting. The mIoU (red line) shows a steady increase, reaching a final value of 67.5%.

This metric highlights the model’s capability to effectively segment structural elements in indoor environments, addressing challenges like occlusions and repetitive patterns. Together, these trends validate the StructScan3D v1 dataset’s utility and the D-Former model’s effectiveness in handling complex indoor scenes.

**Figure 7:**
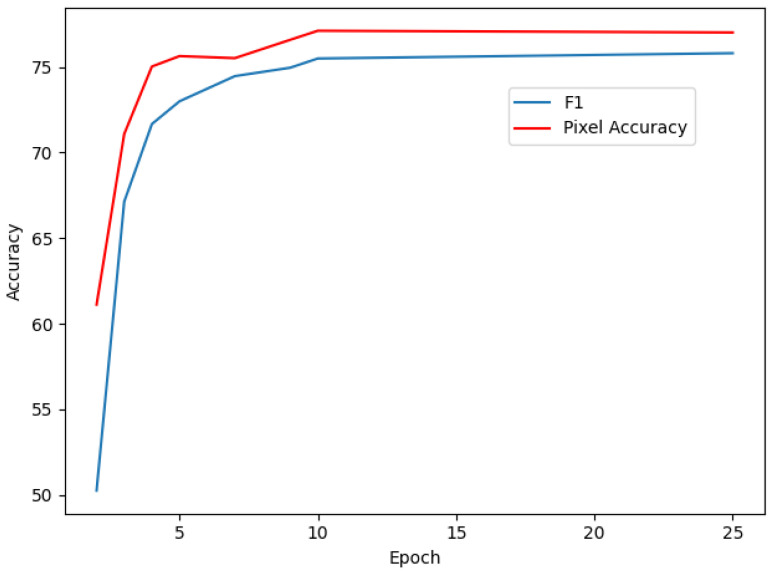
F1 Score (blue) and Pixel Accuracy (red) trends during D-Former training on StructScan3D v1.

The evaluation of the F1 Score and Pixel Accuracy during the training process is presented in Figure 7, where the F1 Score (blue line) rises sharply in the early epochs and stabilizes around 75%, indicating a strong balance between precision and recall, especially for challenging elements like doors and windows. The Pixel Accuracy (red line) improves consistently, surpassing 78% by the end of training, showcasing the model’s reliability in correctly labeling a majority of pixels.

These metrics underline the D-Former model’s ability to adapt to diverse indoor scenes with varying complexities and structural layouts, further emphasizing the robustness of the StructScan3D v1 dataset in enabling detailed and accurate semantic segmentation tasks.

We conducted separate training sessions for D-Former using identical settings on NYU Depth v2, SUN RGB-D, and StructScan3D v1, followed by the computation of the Mean IoU (1) for each model. To assess the effectiveness of the proposed dataset in enhancing semantic segmentation tasks, we benchmarked the D-Former model against widely used RGBD datasets, including NYU Depth v2 and SUN RGB-D.

The results presented in Table 4 clearly show that the D-Former model, with approximately 29.5M parameters, achieves significantly higher performance on our proposed dataset, with an mIoU of 67.5%, compared to 55.6% on NYU Depth v2 and 51.2% on SUN RGB-D, despite using the same model parameters and input resolution. Additionally, the model demonstrates strong consistency across other key metrics. The F1 Score stabilizes at 77% after 10 epochs as shown in Figure 7, reflecting balanced precision and recall in segmentation tasks. Pixel Accuracy reaches over 80% within the first few epochs and remains consistent throughout the training, highlighting the model’s robustness in accurately predicting pixel-level classes. We understand that the higher Mean IoU achieved on the StructScan3D v1 dataset might be due to the simpler scenario of segmenting only structured elements as opposed to the more complex task of segmenting 40 classes in the NYU Depth v2 dataset. The fewer classes (7) in the StructScan3D v1 dataset make it easier for the model to distinguish between different elements, leading to better performance.

To further investigate this, we conducted another experiment to compare the results of our dataset with multiple models. This additional experiment aimed to provide a more comprehensive evaluation and to ensure that the observed performance was not solely due to the simpler scenario but also reflected the robustness of the model across different datasets. To this end, we compare D-Former against two state-of-the-art RGB-D segmentation models: TokenFusion [30] and Gemini [31], both trained on StructScan3D. These models serve as additional benchmarks, providing an objective assessment of D-Former’s strengths and limitations in complex indoor environments.

The evaluation results presented in Table 5 indicate that D-Former achieves significantly higher performance on our proposed dataset, with an mIoU of 67.5%, compared to 55.6% on NYU Depth v2 and 51.2% on SUN RGB-D, despite using the same model parameters and input resolution. Additionally, the model demonstrates strong consistency across other key metrics. The F1 Score stabilizes at 77% after 10 epochs, reflecting balanced precision and recall in segmentation tasks. Pixel Accuracy reaches over 80% within the first few epochs and remains consistent throughout the training, highlighting the model’s robustness in accurately predicting pixel-level classes.

#### Qualitative Results

**Figure 8:**
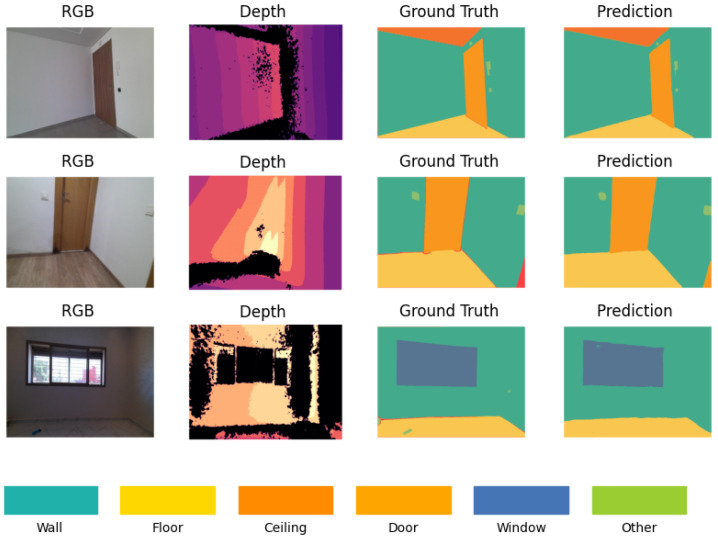
Examples of RGB images, depth maps, ground truth segmentation masks, and predicted segmentation outputs using D-Former model.

The qualitative analysis offers a visual assessment of the D-Former model’s segmentation performance by comparing the predicted segmentation maps against the ground truth annotations for RGB-D inputs. Representative results are presented in Figure 8, showcasing three selected examples from the StructScan3D v1 dataset. The RGB images provide the visual context of the scene, while the depth maps add spatial differentiation, enabling the model to segment elements more accurately in environments with clutter or uniform colors. This combination of RGB and depth data is particularly essential in distinguishing structural elements that share similar visual characteristics but differ in spatial position. The model demonstrates strong performance in segmenting large structural elements such as walls, floors, and ceilings, where boundaries are well-defined. The integration of depth data enhances the model’s ability to handle surfaces that are visually uniform in RGB but differ in depth.

**Figure 9:**
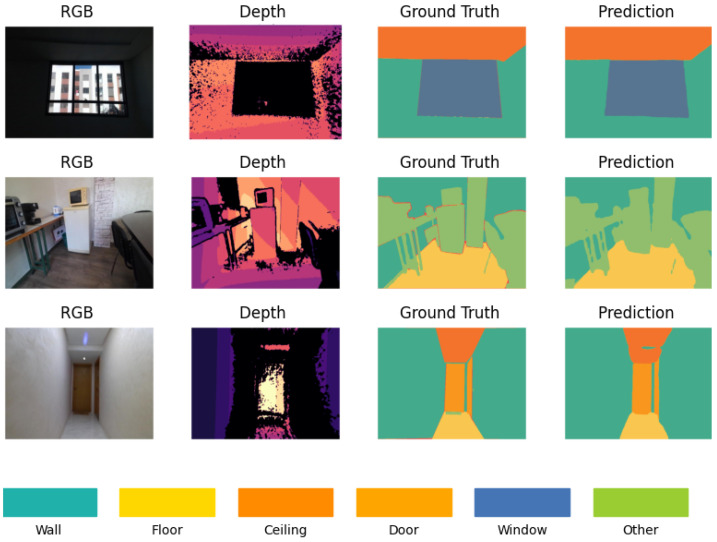
Examples of RGB Images, depth maps, ground truth segmentation masks, and predicted outputs using the D-Former model in complex indoor scenes with occlusions, diverse lighting, and material properties.

However, challenges persist for smaller building elements, such as doors and windows, which occasionally show discrepancies between the predicted segmentation and the ground truth, particularly in cases with occlusions or complex layouts as shown in Figure 9. Scenes with repetitive patterns or low lighting also introduce slight misclassifications, emphasizing areas for potential improvement. Overall, the qualitative results confirm the effectiveness of the StructScan3D v1 dataset in training robust models and highlight the D-Former model’s capacity to capture structural complexity, making it an invaluable resource for applications in BIM, indoor scene reconstruction, and robotics.

### 4.5. Discussion

The quantitative evaluation revealed that the D-Former model achieved an mIoU of 67.5%, demonstrating its capability to accurately segment structural elements by leveraging the rich depth and RGB information provided by the StructScan3D v1 dataset; additionally, the model attained a stable F1 Score of 77%, indicating balanced precision and recall in identifying and classifying structural components. Pixel Accuracy surpassed 80% early in training and remained consistent, reflecting the model’s robustness in assigning correct pixel-level labels. This finding underscores the dataset’s potential to support robust learning in models designed for complex architectural environments.

One of the notable strengths observed was the D-Former model’s ability to accurately segment large structural elements, such as walls, floors, and ceilings, with well-defined boundary delineation. This strength can be attributed to the high-quality depth annotations in StructScan3D, which enhance segmentation in visually challenging areas, including uniform surfaces or regions with overlapping objects. These results confirm the dataset’s capacity to handle diverse structural scenarios effectively. However, as StructScan3D v1 represents an initial benchmark dataset, certain limitations must be acknowledged. In particular, smaller structural elements and materials with complex reflectivity properties remain challenging for current models. Recognizing these constraints provides a more nuanced interpretation of the results and emphasizes the need for future dataset expansions to address these more intricate scenarios.

However, the experiments also highlighted certain limitations. Smaller or occluded elements, such as doors and windows, posed challenges for the model, often resulting in misclassifications. This limitation points to the need for enhanced annotation granularity and model optimization for finer-scale features.

Furthermore, the model struggled in scenes with highly repetitive patterns and low-light conditions, which introduced segmentation errors. Addressing these challenges could involve improving dataset diversity, refining annotations, or exploring hybrid model architectures that integrate the strengths of transformers and convolutional networks.

These findings suggest that while StructScan3D v1 has demonstrated significant value for semantic segmentation, future iterations of the dataset and model enhancements could further optimize performance, particularly for complex and edge-case scenarios. In this regard, an interesting direction for future work would be the application of knowledge distillation methods [32,33], where a smaller segmentation network is trained under the supervision of a larger, more capable network. Such an approach could improve the generalization ability of lightweight models while ensuring computational efficiency, making them more suitable for real-world deployment over the proposed dataset.

## 5. Conclusions

This paper introduced StructScan3D v1, a new RGB-D video dataset meticulously designed to address the challenges of semantic segmentation in indoor environments, particularly within the BIM context. Captured using the Kinect Azure sensor, StructScan3D v1 offers high-quality RGB and depth data, with comprehensive annotations across six structural classes: walls, floors, ceilings, doors, windows, and miscellaneous elements.

Using the D-Former model alongside TokenFusion and Gemini as baselines, StructScan3D v1 demonstrated strong performance, achieving a highest mIoU of 67.5% with D-Former. This result highlights the dataset’s robustness in enabling accurate segmentation tasks, particularly for building elements. The dataset’s high-quality annotations and precise RGB-D alignment contribute significantly to this performance. However, challenges remain in segmenting smaller or occluded features, which represent areas for future exploration.

This study evaluated StructScan3D v1 with multiple state-of-the-art segmentation models, providing a comprehensive benchmark for future research. Future efforts will involve exploring techniques such as knowledge distillation to enhance performance further and expanding the dataset to include more complex, cluttered, and large-scale environments.

To ensure broader applicability, future directions will address limitations in sensor performance, particularly in low-light environments and on reflective or transparent surfaces. Furthermore, we plan to integrate advanced annotation tools and methods to enhance annotation quality for challenging scenarios. Although the Kinect Azure sensor is not as precise as high-end laser scanners, its accuracy remains sufficient for detecting main structural elements such as walls, ceilings, floors, doors, and windows. This first version of the dataset aims to support automatic semantic segmentation rather than high-precision geometric modeling, and the proposed framework can later be extended using data from more accurate sensors.

StructScan3D v1 addresses a critical gap in the field by providing an annotated RGB-D dataset specifically tailored for semantic segmentation in BIM-related tasks. Designed under realistic acquisition and lighting conditions, it supports the training, validation, and benchmarking of deep learning models in structured indoor environments. While this first version focuses on core architectural elements, it establishes a solid foundation for future extensions that will incorporate a wider range of scenes and structural components—including industrial, educational, and medical settings. These future developments aim to broaden generalization and support increasingly complex segmentation scenarios.

Beyond semantic segmentation and BIM reconstruction, StructScan3D v1 offers practical utility across different domains such as the following:

**Digital construction and facility management**: The dataset can be used to update as-built BIM models by detecting semantic changes in building layouts, and to support renovation planning and maintenance tasks through improved understanding of spatial organization.

**Robotics and indoor intelligence**: StructScan3D enables context-aware navigation and object localization by providing semantically rich scene information, which can enhance the performance of service robots and facilitate human–robot interaction in indoor spaces.

**Immersive technologies and simulation**: The dataset contributes to the generation of accurate and labeled indoor models for augmented and virtual reality applications, and can support emergency preparedness by enabling detailed spatial modeling for evacuation and safety simulations.

By bridging the gap between academic research in RGB-D semantic segmentation and applied scenarios, StructScan3D v1 serves as a valuable and extensible benchmark for researchers, developers, and industry practitioners involved in indoor modeling and digital built environment technologies.

To foster collaboration and enable reproducibility in the research community, the StructScan3D v1 dataset is publicly accessible through https://github.com/ishraqrc/StructScan3D.git, accessed on 1 April 2025.

## Figures and Tables

**Table 1:** Overview of the most popular RGB-D datasets.

Dataset	Description	Annotated Scenes	Number of Classes	Main Limitations	Years
NYU Depth v2 [10]	Indoor scenes with RGB-D images, primarily single-frame focused	1449 frames	40	No sequential annotation for spatio-temporal models	2012
SUN RGB-D [13]	Indoor scenes dataset with RGB-D images for semantic segmentation	10,335 images	37	Lacks temporal dynamics across frames	2015
ScanNet [18]	RGB-D dataset for indoor 3D reconstruction	2.5 M frames across 1513 scenes	20	Sparse labels across sequences; not dense enough for temporal tasks	2017
Matterport3D [11]	Panoramic RGB-D dataset for 3D indoor scene reconstruction and segmentation	10,800 panoramic views	21	Focuses on panoramic images, limiting usability for pixel-wise segmentation	2017
InteriorNet [20]	Large-scale synthetic dataset for RGB-D and 3D indoor scene understanding	1 M+ synthetic frames	30	Synthetic nature, domain gaps compared to real-world datasets	2018
SceneNet RGB-D [19]	Synthetic dataset with densely annotated RGB-D frames for indoor environments	5 M synthetic frames	13	Entirely synthetic; lacks diversity and realism of real-world environments	2017
Hypersim [21]	High-quality synthetic dataset with photorealistic indoor scenes and RGB-D data	461 scenes	80	Synthetic; computationally heavy to process	2021

**Table 2:** Metadata summary of scene structure and image distribution.

Scene	Description	Number of Sub-Scenes	Total Images
Scene 1	Residential apartment	22	443
Scene 2	Residential apartment	6	663
Scene 3	Office space	1	487
Scene 4	Office space	1	293
Scene 5	Office space	1	293
Scene 6	Office space	1	123
Scene 7	Office space	1	156
Scene 8	Office space	1	89
Scene 9	Office space	1	52

**Table 3:** Detailed characteristics of the dataset.

Dataset Characteristics	Details
Total Scenes	9
Scene Types	Residential, Office
Dataset Split	Training Set: 5 scenes (Residential: 2, Office: 4);Testing Set: 3 scenes (Residential: 1, Office: 2)
Total Images	2594
Training Images	1910 images
Testing Images	684 images
Resolution	480 × 640 pixels
Annotation	Semantic Segmentation
Classes	6 (Ceiling, Door, Floor, Wall, Window Other)
Class Distribution (Overall)	Ceiling: 549, Door: 528, Floor: 1056, Wall: 1644, Window: 590, Other: 1779
Sensor Used	Kinect Azure
Data Format	RGB images with corresponding depth maps
Annotation Tool	Semi automatic/Manuel
Dataset Versions	Version 1 (as of 21 November 2024)
Key Features	Temporal and spatial annotations for semantic segmentation, scene understanding, indoor modeling

**Table 4:** Comparative performance of D-Former with 29.5M parameters across different datasets.

Dataset	Number of Classes	Input Resolution	Mean IoU (%)
NYU Depth v2	37	480 × 640	55.6
SUN RGB-D	40	530 × 730	51.2
StructScan3D v1 dataset (ours)	7	480 × 640	67.5

**Table 5:** Performance comparison across models.

Dataset	Model	Parameters (M)	Backbone	Mean IoU (%)
StructScan3D-v1	Tokenfusion	26.01	mit_*b*_2	65.3
	Gemini fusion	22.19	mit_*b*_1	65.9
	D-Former	29.5	SegFormer Base (B2)	67.5

## Data Availability

The StructScan3D dataset used in this study is publicly available at: https://github.com/ishraqrc/StructScan3D, accessed on 1 April 2025.
